# Progress and Disparities in Lung Cancer Screening in Japan: A Bayesian Analysis Toward Achieving Health Japan 21 Targets

**DOI:** 10.3390/cancers18101498

**Published:** 2026-05-07

**Authors:** Takao Suzuki, Hasan Jamil, Aminu Kende Abubakar, Tshewang Gyeltshen, Hellen Wairimu Babu, Phuong The Nguyen

**Affiliations:** 1 Research Center for Health Policy and Economics, Hitotsubashi Institute for Advanced Study (HIAS), Hitotsubashi University, Kunitachi, Tokyo 186-8601, Japan; 26dp003@slcn.ac.jp (T.S.); 24dp002@slcn.ac.jp (H.J.); tgyeltshen@g.ecc.u-tokyo.ac.jp (T.G.); 24dp001@slcn.ac.jp (H.W.B.); 2 Graduate School of Public Health, St. Luke’s International University, Chuo, Tokyo 104-0045, Japan; 3 Division of Population Data Science, National Cancer Center Institute for Cancer Control, Chuo, Tokyo 104-0045, Japan; 4 Global Health Policy Department, School of International Health, Graduate School of Medicine, University of Tokyo, Bunkyo, Tokyo 113-8654, Japan

**Keywords:** lung cancer screening, Bayesian forecasting, prefectural variation, sex disparities, health Japan 21, Japan

## Abstract

**Background**: Japan’s national health promotion plan, *Health Japan 21*, aims to achieve 60% lung cancer screening coverage by 2028. This study projected screening uptake through 2028 and estimated the probability of achieving this target at the national and prefectural levels, stratified by gender. **Methods**: We analyzed lung cancer screening in Japan using the Comprehensive Survey of Living Conditions data (2013, 2016, 2019, 2022) for adults aged 40–69. Bayesian linear regressions estimated trends in logit-transformed screening coverage, with projections through 2028. We estimated annual changes, the probability of reaching 60% by 2028, and the first year in which the probability of target attainment exceeded 80%. **Results**: National screening coverage increased from 42.3% in 2013 to 49.7% in 2022, with an annual percentage-point change of 0.83 (95% CrI: −0.20 to 1.80). At this rate, the probability of reaching 60% by 2028 is 12.2%. Only 11 of 47 prefectures are projected to meet the target, while 4 (Kyoto, Kumamoto, Kagoshima, and Okinawa) are unlikely to reach it. Sex disparities were observed, with the probability of achievement at 25.4% for men versus 7.1% for women. The female annual change (0.99) exceeded the male annual change (0.65). **Conclusions**: Lung cancer screening participation varies significantly across regions and between genders, reflecting uneven progress toward the national goal. If current trends continue, achieving 60% coverage by 2028 will be challenging for most prefectures. Policy and system reforms could speed up uptake by focusing on prefectures and populations with the lowest participation rates.

## Simple Summary

Lung cancer remains a major cause of illness and death in Japan, but the benefits of screening depend on whether enough people participate. Japan has set a national goal of achieving 60% lung cancer screening coverage by 2028, yet progress may vary across prefectures and between women and men. In this study, we used national survey data to project future screening participation and estimate the likelihood that each prefecture will meet the national target. We found that, under current trends, reaching the target by 2028 will be difficult, with clear differences across areas and by sex. These findings provide an early warning for policymakers and researchers by showing where progress is lagging and where stronger efforts may be needed to improve screening uptake and reduce inequalities.

## 1. Introduction

Lung cancer has been the world’s most diagnosed cancer (12.4% of all new cancer cases) and the leading cause of cancer death (18.7% of cancer deaths) since 2022 [1]. In Japan, there were 124,531 lung cancer cases in 2021, 75,569 deaths in 2024, and a 34.9% 5-year relative survival rate (2009–2011) [2]. The burden of lung cancer varies substantially by sex and across prefectures [2]. We previously projected that lung and colorectal cancers would remain among the leading burdens of incidence and mortality in Japan through 2054 [3,4].

To reduce lung cancer mortality, early detection is essential, and regular screening plays a vital role. However, detecting lung cancer at an early stage remains challenging because the sojourn time during which the asymptomatic disease is detectable by chest radiography screening has been estimated at approximately 5.5 months in the general population [5] and up to 2.2 years among male heavy smokers screened with chest radiography plus sputum cytology [6]. This limited detection window makes early diagnosis difficult, underscoring the importance of appropriately timed screening programs. Modeling studies suggest that shorter screening intervals increase lead time [6]. Japan mandates annual lung cancer screening for all adults aged 40 years and older under the Health Promotion Act [7]. The MHLW has issued detailed guidelines specifying annual chest radiography for all individuals aged 40 years and older, with the addition of sputum cytology for those aged 50 years or older who are heavy smokers (Brinkman Index ≥600) [8]. According to the 2022 CSLC, the national uptake rate of lung cancer screening in Japan was 49.7%. Uptake was lower among women (46.4%) than men (53.2%), and current participation levels remain insufficient to meet recommended targets [9].

The Health Promotion Act also provides the legal basis for a wide range of health promotion measures. Article 7 stipulates that the MHLW is to establish a basic policy to comprehensively advance improvements in people’s health [7]. On this basis, the Japanese government has implemented the National Health Promotion Movement in the 21st Century (*Health Japan 21*) to extend healthy life expectancy and reduce health disparities, with specific targets established within each program cycle. The current program is the third term, scheduled to run from 2024 to 2035 [10]. *Health Japan 21* sets explicit targets for cancer screening uptake, including a national goal of achieving a 60% uptake rate for all cancer screenings, including lung cancer, by 2028 [10]. In the latest report, both the national and prefectural governments have developed policies to reach this target. However, the current uptake rates remain below the 60% benchmark [9].

Improving lung screening uptake to achieve the third-term *Health Japan 21* target requires understanding prefectural and gender-specific trajectories and identifying populations to prioritize for intervention. Our recent analysis of *Health Japan 21* tobacco targets found pronounced regional disparities in progress [11]. The same challenges can impede equitable gains in cancer screening uptake. Forecasting Lung screening uptake and the probability of meeting the target is critical for evaluating policy feasibility and guiding strategies to address regional and population-level gaps. Studies that integrate national trends, regional variation, and gender disparities in lung cancer screening remain limited. This study aimed to: (1) project cancer screening uptake rates across all 47 prefectures in Japan through 2028, (2) estimate the probability of achieving the 60% national target at both the national and prefectural levels, and (3) quantify regional and gender-based disparities in current uptake and projected progress. Given the limited time left until 2028, this study addresses the need to tackle persistently low screening uptake rates and to develop additional policies to accelerate progress toward the national goal.

## 2. Materials and Methods

### 2.1. Study Design and Data Source

This prefecture-level longitudinal analysis tracked lung cancer screening uptake across Japan over a ten-year observation period (2013–2022). Screening participation rates for residents aged 40 to 69 were obtained from the official statistics of Japan website, which reports estimates from the Comprehensive Survey of Living Conditions (CSLC) [12,13]. We focused on this age group to align with *Health Japan 21*. The CSLC is a large-scale household survey administered every three years by the Ministry of Health, Labour and Welfare (MHLW). Four survey cycles (2013, 2016, 2019, 2022) were included in the analysis. Data from 2007 and 2010 survey waves were excluded due to a structural break in screening trends between 2010 and 2013, coinciding with Japan’s 2012 revision of the Basic Plan to Promote Cancer Control, which mandated municipal screening with individual invitation systems. This yielded four data points per prefecture-gender unit.

### 2.2. Outcome Measures

The primary outcome was the percentage of age-eligible adults who reported undergoing lung cancer screening within each survey year. Three derivative indicators were computed: the Bayesian posterior probability of achieving the 60% uptake benchmark by 2028, the average annual change in participation, measured in percentage points, and the calendar year when the probability of target attainment first exceeded 0.80. Gender disparity was defined as the difference between male and female rates (M–F diff), in percentage points, and was calculated for both screening coverage and the posterior probability of achieving the 60% target by 2028. Positive values indicate higher rates among men.

### 2.3. Statistical Approach

Coverage percentages were logit-transformed prior to modeling. For each prefecture-gender combination, a Bayesian linear regression was fit with centered calendar year as the sole covariate. Centering on the baseline survey year reduced the correlation between the intercept and slope parameters. Weakly informative priors were specified. Posterior sampling was performed via Markov chain Monte Carlo with three chains, each run for 12,000 iterations. The initial 2000 draws were discarded as burn-in, and the remaining 30,000 draws (pooled across chains) formed the basis for inference. Convergence was confirmed by trace-plot inspection. Predictions on the probability scale were obtained by applying the inverse-logit transformation to the sampled linear predictors [14,15]. The training window was restricted to 2013–2022 to exclude a structural break attributable to the 2012 Basic Plan revision.

Posterior predictive distributions for 2028 coverage were generated by extending each fitted trend forward two triennial survey intervals (six years from the final observation of 2022). The fraction of posterior draws exceeding the 60% threshold defined the probability of achieving the target. A prefecture was classified as on track to meet the national goal if this exceedance probability was 0.80 or greater. Progress velocity was summarized as the model-estimated annual gain in coverage, expressed on the original percentage scale.

The annual percentage change (APC) was obtained from each model’s posterior samples. For each draw, baseline coverage was computed as the inverse logit of the intercept (α), and coverage one year later as the inverse logit of α + β, where β is the annual slope; the difference between these two values, in percentage points, is the APC for that draw. Point estimates and 95% credible intervals were taken as the mean and 2.5–97.5th percentiles of the APC distribution.

Out-of-sample accuracy was evaluated by training models on the first three post-structural-break survey waves (2013, 2016, 2019) and generating predictions for the held-out fourth wave (2022) [16]. Discrepancies between forecasted and observed values were quantified using five metrics: mean absolute deviation, root-mean-squared deviation, systematic bias, mean absolute percentage deviation, and the empirical coverage of 95% posterior intervals.

## 3. Results

### 3.1. Observed and Projected Screening Uptake by Prefecture and Sex (2013–2028)

Table 1 presents baseline screening coverage in 2013, observed coverage in 2022, and projected coverage for 2028 (95% CrI). In 2013, the national screening coverage was 42.3%, well below the 60% target. Coverage ranged from 32.3% in Osaka to 60.0% in Yamagata, which had already attained the national target. The gender gap was 10.1 percentage points (47.5% for men versus 37.4% for women). Men reported higher coverage in all 47 prefectures, with the largest differences in Hyogo (14.7 percentage points), Kanagawa (14.6), and Shiga (13.8).

### 3.2. Screening Uptake in 2022

By 2022, national screening coverage had increased to 49.7% (53.2% for men, 46.4% for women), an improvement of 7.4, 5.7, and 9.0 percentage points, respectively, since 2013. The gain was larger among women than men, narrowing the national gender gap from 10.1 to 6.8 percentage points. Men continued to report higher coverage across all 47 prefectures, with the gap ranging from 0.6 percentage points in Okinawa to 14.4 percentage points in Nara. Total coverage ranged from 40.7% in Hokkaido to 69.0% in Yamagata, and only four prefectures (Miyagi, Yamagata, Niigata, and Yamanashi) had reached the 60% target. The level of improvement varied from 5.0 percentage points in Hokkaido to 9.4 in Osaka. Sex-specific ranges remained broad: 44.9% (Okinawa) to 69.8% (Yamagata) for men, and 34.9% (Hokkaido) to 67.6% (Yamagata) for women (Table 1).

### 3.3. Annual Percent Change and Projections to 2028

The annual percent change (APC) was estimated for each prefecture using data from 2013 to 2022. All prefectures showed upward trends for total, male, and female populations, but many 95% credible intervals included zero, meaning that trends were not statistically distinguishable from no change in several cases. The nationwide APC was 0.83 (95% CrI: −0.20, 1.80) for the total population, 0.65 (−0.28, 1.56) for men, and 0.99 (0.03, 1.92) for women. The female APC exceeded the male APC, meaning that women’s coverage has been increasing faster than men’s in the post-2013 period. For total coverage, Gifu showed the largest increase (1.24 [−0.02, 2.40]), and Okinawa the smallest (0.37 [−0.42, 1.14]). Among men, Gifu had the highest APC (1.31 [0.01, 2.58]) and Okinawa the lowest (0.22 [−0.66, 1.10]). Among women, Tokyo had the highest APC (1.48 [0.23, 2.62]) and Yamaguchi the lowest (0.41 [−0.27, 1.05]) (Table 2).

**Figure 1:**
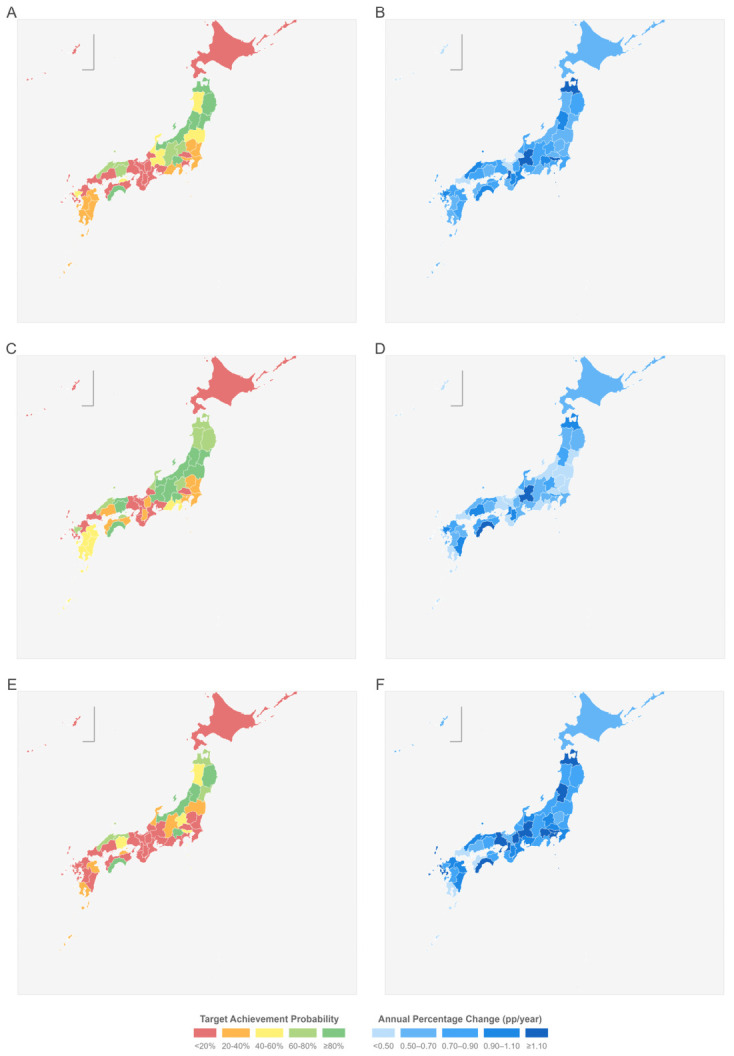
Projected lung cancer screening coverage and trends by prefecture and sex, 2028. Geographic variation in lung cancer screening projections across 47 Japanese prefectures. Panels show total population (**A**,**B**), male (**C**,**D**), and female (**E**,**F**). Left column (**A**,**C**,**E**): Probability of achieving ≥60% coverage by 2028, ranging from red (<20%, low) to green (≥80%, high). Right column (**B**,**D**,**F**): Annual percentage change (APC) in coverage, expressed in percentage points per year, displayed as the posterior mean per prefecture. Categories are <0.50 (light blue), 0.50–0.70, 0.70–0.90, 0.90–1.10, and ≥1.10 (darkest blue). All posterior-mean APCs are positive across the 47 prefectures × 3 population groups shown; for several prefectures the 95% credible interval includes zero, as reported in Table 2.

Bayesian projections indicate that only 11 prefectures (Aomori, Iwate, Miyagi, Yamagata, Niigata, Toyama, Yamanashi, Nagano, Tottori, Okayama, and Kochi) are projected to meet the *Health Japan 21* target of 60% coverage by 2028. A further 10 prefectures are projected to achieve 60% by 2040, while 4 prefectures (Kyoto, Kumamoto, Kagoshima, and Okinawa) are unlikely to reach the target within the foreseeable future. Hokkaido, with the slowest projected trajectory, is estimated to reach the target around 2081. Coverage growth has decelerated across most of the country since the rapid gains of the 2010–2013 period (Table 1 and Table 2; Figure 1).

The geographic distribution of achievement probabilities and annual percentage change by sex is shown in Figure 1, which reveals that on-track prefectures are concentrated in the Tohoku and Chubu regions, while Kinki and southern Kyushu lag furthest behind.

### 3.4. Gender Disparities in Target Achievement

**Figure 2:**
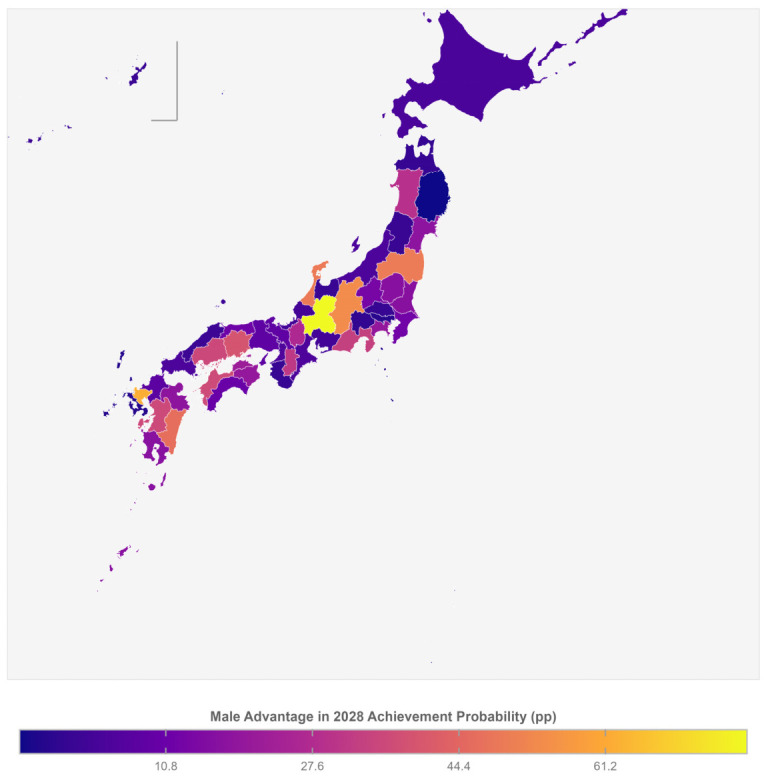
Sex disparity in probability of achieving ≥60% lung cancer screening coverage by 2028. Map shows the male–female difference in achievement probability (percentage points) for each prefecture. Higher values indicate a larger male advantage. Gifu shows the largest disparity (77.8 percentage points), while Iwate shows near-parity.

Figure 2 shows gender differences in target achievement. Whereas 11 prefectures were projected to reach 60% coverage for the total population by 2028, this number was 12 for men but only 8 for women. The nationwide probability of achievement was 25.4% for men versus 7.1% for women, a gap of 18.3 percentage points. At the prefectural level, the largest gender gap in achievement probability was in Gifu (77.8 percentage points: men 91.3% versus women 13.5%), followed by Saga (61.4 percentage points) and Nagano (54.8 percentage points). Defining “large disparity” as a male–female difference of 15 percentage points or more in achievement probability, 22 prefectures met this criterion (Table 2). By contrast, Iwate showed near-parity between men and women. The projected male–female difference in coverage is narrowing, from 10.1 percentage points at baseline (2013) to 6.8 in 2022 and a projected 4.2 by 2028, consistent with women’s faster rate of improvement in the post-2013 period (Table 1). The geographic distribution of gender disparities in achievement probability is shown in Figure 2, where Gifu, Saga, and Nagano stand out as having the widest male advantage, while Tohoku prefectures in the north show near-parity.

### 3.5. Model Validation

Out-of-sample validation was performed by training models on 2013–2019 data and predicting held-out 2022 values. Across 138 prefecture-gender pairs, the mean absolute error was 3.49 percentage points with a positive bias of +3.15 percentage points, and 95% credible interval coverage was 100%. Restricting the training window to post-2013 data outperformed full-period models (mean absolute error approximately 10 percentage points), confirming the presence of the 2010–2013 structural break. Full validation results are presented in Table S1, with predicted versus observed values shown in Figure S1 and prefecture-level validation performance in Figure S2.

## 4. Discussion

Early detection improves the prognosis of lung cancer. The Japanese government provides population-based lung cancer screening to adults aged 40 and older and has set a target of achieving a 60% uptake rate by 2028. Our projection, based on post-2013 trends that exclude the one-time structural gains associated with the 2012 Cancer Control Plan revision, suggests that most prefectures will not meet the 60% target by 2028. Only 11 prefectures are projected to achieve the benchmark on schedule, while 4 are unlikely to reach it within the foreseeable future. Large gender and regional disparities in the timing and likelihood of achievement remain. These findings point to the need for renewed and intensified policy intervention to accelerate progress toward the national goal.

Several factors have been identified as barriers to lung cancer screening participation, including emotional factors such as fear of diagnosis and structural barriers such as cost and access [17,18]. In Japan, one factor contributing to persistently low uptake is the complexity of the screening delivery system [19]. Cancer screening in Japan can be classified into three distinct programs: (1) legally mandated municipality-based screening, (2) workplace-based screening by insurers or employers, and (3) opportunistic *Ningen Dock* check-ups [19,20,21]. The Health Promotion Act (2008) provides a legal basis only for municipality-based programs, yet more than 50% of the population utilizes workplace-based screening, which lacks a formal statutory foundation [19]. There is no integrated national system for consolidating screening participation information. Identification of eligible individuals, documentation of participation, and outreach to non-participants are conducted independently by each screening provider [19]. This fragmentation is compounded by the large number of health insurers, each offering distinct services. The level of promotion and quality of service vary across insurers and employers, with some actively promoting participation and others not [22]. Because these services are offered on a voluntary basis by employers, their quality is variable. Participation in lung cancer screening increases with company size for both sexes, and non-regular workers consistently show the lowest levels [23]. A large proportion of the Japanese population undergoes screening through workplace-based programs organized by insurers or employers, yet these programs lack a clear legal foundation [21]. The coexistence of multiple, fragmented screening programs complicates coordination and may impede centralization of cancer control efforts. Fragmentation in screening governance is not unique to Japan. A 2025 policy mapping of low-dose CT-based lung cancer screening across the 27 European Union Member States and three non-EU European countries found that only seven EU countries had implemented an LDCT-based program, with wide heterogeneity in eligibility criteria, funding, and implementation status [24]. Japan’s experience parallels a broader international pattern in which screening policy intentions outpace harmonised delivery. These findings and our projections suggest the need for the central government to establish a unified governance framework for cancer screening.

The gender disparities in target achievement probabilities projected in this study warrant attention. The achievement probability is 12.2% for the total population, 25.4% for men, and 7.1% for women, a gap of 18.3 percentage points between men and women. The female APC (0.99) exceeded the male APC (0.65) in the post-2013 period, and the gender gap in coverage is narrowing. This may reflect the broader reach of the individual invitation systems mandated by the 2012 Cancer Control Plan revision, which extended screening outreach beyond the workplace-based programs that historically favored male employees. A possible explanation for this disparity is the reliance on workplace-based screening systems. Workplace-based screening primarily targets full-time employees, most of whom are men, while women are more likely to be in non-regular employment with poorer access to screening services. The Cabinet Office survey reported higher screening uptake rates among full-time employees than among non-regular employees for both sexes [25]. The uptake rate among full-time employed women was nearly twice that of homemakers [25]. In Japan, large gender differences remain in employment rates and the proportion of individuals in full-time employment [26]. Women are more likely to hold non-regular jobs or be full-time homemakers, which may increase their likelihood of missing workplace screening opportunities.

At the individual level, women are generally considered to have greater health awareness and higher interest in preventive health behaviors than men [27,28]. Smoking has been identified as a major barrier to screening participation, and a negative association between smoking behaviour and screening uptake has been consistently reported [29,30]. Sex differences in smoking prevalence are also observed in Japan, with approximately 28% among men and 9% among women [31]. Additionally, heavy smoking (20 or more cigarettes per day) was significantly associated with decreased screening rates for lung, stomach, and colorectal cancers among men, but only for colorectal cancer among women [32]. This suggests that men might be expected to have higher non-participation at the individual level. The persistence of lower screening participation among women, therefore, suggests that institutional and structural factors, rather than individual preferences alone, play a large role. In recent years, the increasing incidence of lung adenocarcinoma among never-smoking women has emerged as a major public health concern [33]. Women should therefore be recognized as a high-risk population for lung cancer, and targeted preventive strategies should not be neglected.

Our projections showed clear regional disparities. Some prefectures had already achieved a screening uptake rate of 60% by 2022, whereas Hokkaido is projected to reach this level around 2081, which is effectively unattainable at current growth rates. Although inter-prefectural disparities pose a significant public health challenge, research to reduce these gaps remains limited. Based on current trends, 36 prefectures are projected to have difficulty achieving the target by 2028, with 4 prefectures (Kyoto, Kumamoto, Kagoshima, and Okinawa) unlikely to reach it at all under current trajectories. This suggests that more intensive and target-specific strategies are required, particularly in the lowest-performing prefectures. Geographic disparities in lung cancer screening access are well documented internationally. In the United States, rural counties show consistently higher lung cancer incidence and mortality than urban counties together with lower screening uptake, and complementary strategies such as AI-based screening tools have been proposed to help narrow these gaps [34]. Few studies have examined the determinants of inter-prefectural disparities in cancer screening in Japan. Without identifying the underlying factors, it is difficult to develop target-specific interventions.

Several prefectures carry credible intervals so wide that their projected coverage is statistically consistent with both meeting and substantially missing the 60% target. Such estimates are best read as monitoring priorities rather than definitive forecasts, and their classification will sharpen as additional CSLC waves become available.

Socioeconomic status (SES) is a key factor influencing participation in cancer screening, with lower uptake often observed among individuals with lower SES [35,36]. Even at the population level, areas with lower SES had higher lung cancer incidence but lower screening detection rate [37]. Consistent with these findings, our study observed higher uptake rates in prefectures with relatively higher SES, whereas achievement of the target was projected to be more challenging in prefectures with lower SES, suggesting an association with socioeconomic context. Other factors may contribute to inter-prefectural disparities in uptake rates, including differences in employment status, the capacity and accessibility of screening facilities, financial support for cancer screening, and variation in the content and implementation of Prefectural Cancer Control Promotion Plans. Without identifying the reasons why screening uptake rates remain low in certain prefectures, it is not possible to implement effective countermeasures. This study focuses on projections of uptake rates and target achievement probabilities for 2028 and therefore cannot assess the determinants of these disparities. To achieve the *Health Japan 21* targets, more effective, evidence-based policy development is required, and further research is needed to generate the evidence to inform such strategies.

While low-dose computed tomography is increasingly becoming the global standard, in Japan, chest X-ray has become established as a modality for lung cancer screening, and some degree of mortality reduction has been suggested [8]. The effectiveness of low-dose computed tomography has been demonstrated mainly among heavy smokers, but the evidence among never-smokers remains insufficient. At present, chest X-ray is considered the only screening modality applicable to the general population and is likely to remain the cornerstone of lung cancer screening in Japan for some time [38,39,40].

Technological advances may complement the policy reforms our findings call for. Recent deep-learning models for chest radiography, the modality used by Japan’s national screening program, have reached high accuracy on multi-class lung-condition datasets [41]. Such tools cannot raise uptake on their own, but they can reduce reader workload and free clinical capacity for outreach to under-screened populations.

A key finding of this study is the deceleration in the growth of screening uptake since the initial surge driven by the 2012 Cancer Control Plan revision. The rapid gains of 2010–2013 (approximately +17.6 percentage points nationwide in three years) have not been sustained. Post-2013 growth has slowed to an APC of only 0.83 percentage points per year. While the initial policy reform was effective in raising coverage from approximately 25% to 42%, its momentum has stalled, and a renewed cycle of policy intervention is needed to close the remaining gap to 60%. Early detection is essential for a favorable prognosis of lung cancer, and achieving the target uptake rates set forth in *Health Japan 21* is necessary for reducing lung cancer mortality. This study found clear gender and regional disparities, but quantitative research on the factors underlying differences in screening participation between men and women and inter-prefectural differences remains limited. The significance of this study lies in its ability to quantify these disparities and the urgency of addressing them.

This study has several limitations. First, screening uptake rates were derived from the CSLC, which relies on self-reported responses. Recall bias is possible: some respondents may not recognize that they underwent screening, while others may inaccurately report participation. The reported rates were not age-adjusted and did not account for differences in population structure across prefectures, which may affect inter-prefectural comparisons. Second, the model was trained on only four triennial survey waves (2013–2022), yielding four data points per prefecture-sex unit. Although the Bayesian framework propagates this uncertainty into wide credible intervals, the small sample size limits the precision of trend estimates. Third, the linear trend assumption on the logit scale implies a constant rate of change throughout the projection period. The model cannot capture acceleration, deceleration, or saturation effects that may occur as coverage approaches 50–60%. If uptake growth slows further, the projections may be optimistic. Conversely, new policy interventions could accelerate progress beyond what the model predicts. Fourth, the 2019–2022 interval includes the COVID-19 pandemic, which may have temporarily depressed screening participation. If the pandemic effect was transient, the estimated post-2013 trend may understate the underlying growth rate, and projections may be pessimistic. Fifth, the CSLC does not record smoking status at the prefecture-sex level, so we could not stratify projections by smoking history. This matters because smoking is a major determinant of lung cancer risk and shapes screening eligibility under the Brinkman Index ≥600 sputum cytology criterion. The projected uptake rates therefore aggregate over smokers and non-smokers and cannot speak to whether the 60% target is being met in the higher-risk smoking subpopulation. Sixth, the model is descriptive rather than explanatory. It projects trends but does not include covariates such as socioeconomic status, urbanization, or health system capacity, and therefore cannot identify the causes of inter-prefectural or sex-based disparities. Finally, excluding the 2007 and 2010 survey waves was necessary to address the structural break associated with the 2012 Cancer Control Plan revision, but this decision reduced the number of training observations. Sensitivity to this choice was assessed through out-of-sample validation, which confirmed that the restricted training window produced more accurate predictions than the full-period model.

## 5. Conclusions

This study projects the lung cancer screening uptake rate for 2028 based on post-2013 trends, accounting for the structural break introduced by the 2012 Cancer Control Plan revision. The nationwide probability of achieving the 60% target is low, at approximately 12%, and the achievement probability varies across prefectures and genders. The deceleration in screening uptake since 2013 indicates that the momentum generated by the 2012 reform has not been sustained. Structural defects, including decentralized and multi-sectoral health check-up provisions, likely contribute to the remaining disparities. Evidence explaining the factors behind gender and regional disparities is also lacking. To achieve the *Health Japan 21* screening coverage target, renewed and intensified policy interventions are essential, along with further investigation into the causes of disparities and the implementation of systemic reforms. Future research should evaluate how predictive analytics, AI-based risk stratification, and digital health platforms could be integrated into national screening strategies to support uptake and efficient delivery in the lowest-performing regions.

## Figures and Tables

**Table 1:** Lung cancer screening coverage by prefecture and sex: baseline (2013), current (2022), and projected (2028).

	2013 (%)			2022 (%)			2028 Projected, % (95% CrI)		
Prefecture	Total	Men	Women	Total	Men	Women	Total	Men	Women
Nationwide	42.3	47.5	37.4	49.7	53.2	46.4	55.8 (44.5, 66.3)	58.0 (47.4, 68.3)	53.8 (42.5, 64.9)
Hokkaido	35.7	42.1	30.0	40.7	47.1	34.9	45.4 (34.0, 57.0)	51.6 (39.1, 64.6)	40.0 (29.4, 51.3)
Aomori	44.7	49.0	41.6	55.3	57.0	53.6	64.0 (47.5, 79.4)	64.0 (43.6, 80.9)	62.7 (50.7, 73.7)
Iwate	52.3	53.3	51.3	59.0	59.5	58.6	63.3 (53.7, 71.9)	64.4 (43.2, 82.2)	62.4 (54.3, 70.1)
Miyagi	55.1	59.9	50.7	60.0	62.4	56.8	64.4 (48.0, 78.2)	65.3 (51.5, 77.4)	62.4 (42.0, 79.0)
Akita	50.9	54.0	47.9	55.4	58.1	53.7	59.9 (45.4, 72.7)	62.6 (46.4, 76.7)	59.3 (43.5, 72.7)
Yamagata	60.0	62.5	56.6	69.0	69.8	67.6	74.1 (65.2, 81.4)	74.5 (62.8, 83.9)	73.4 (61.3, 83.2)
Fukushima	50.8	55.1	46.2	55.8	59.1	52.0	60.4 (49.8, 70.7)	62.0 (55.6, 68.2)	58.1 (41.5, 74.7)
Ibaraki	44.2	48.3	40.0	50.3	53.2	47.3	56.1 (36.0, 76.1)	57.5 (35.0, 77.0)	54.1 (32.2, 75.0)
Tochigi	47.7	51.7	44.2	52.4	54.7	50.1	57.1 (40.1, 72.7)	58.2 (43.2, 72.1)	55.6 (39.7, 70.7)
Gunma	48.8	51.3	46.0	55.7	57.4	53.6	62.2 (42.7, 79.0)	63.2 (41.1, 82.4)	60.8 (42.2, 76.8)
Saitama	40.2	46.2	34.3	45.9	48.6	43.4	51.6 (36.4, 66.1)	52.2 (37.9, 66.9)	51.6 (36.9, 66.1)
Chiba	45.2	49.2	41.4	52.2	54.6	50.1	57.4 (47.3, 67.3)	58.5 (49.4, 67.1)	56.4 (44.1, 67.8)
Tokyo	40.7	46.4	35.2	50.5	53.0	48.3	58.7 (44.3, 72.1)	58.8 (45.5, 70.8)	58.9 (44.4, 71.9)
Kanagawa	41.8	49.2	34.6	50.0	54.9	45.4	55.7 (47.7, 63.8)	58.1 (48.7, 66.3)	53.6 (44.8, 62.5)
Niigata	54.5	58.3	50.7	60.6	62.7	58.1	64.7 (48.4, 78.7)	65.9 (46.8, 81.6)	63.3 (50.2, 75.0)
Toyama	51.2	55.7	47.6	58.1	59.7	57.4	63.2 (56.1, 69.7)	63.0 (53.5, 71.5)	63.1 (54.8, 70.9)
Ishikawa	47.5	51.8	43.3	54.5	57.6	51.1	60.7 (43.9, 75.3)	63.4 (40.7, 82.0)	57.5 (45.7, 68.2)
Fukui	47.9	51.9	43.8	51.5	54.1	50.0	54.5 (47.6, 61.1)	55.9 (48.5, 63.4)	54.7 (47.0, 62.2)
Yamanashi	54.5	57.6	50.9	62.9	63.6	61.4	68.4 (60.8, 75.2)	67.6 (57.3, 77.4)	68.2 (59.1, 77.0)
Nagano	50.2	54.5	45.8	57.1	61.2	53.3	61.3 (53.2, 68.9)	64.1 (52.0, 75.0)	58.8 (49.2, 67.9)
Gifu	40.9	46.3	35.8	51.9	57.6	46.9	60.6 (47.7, 72.3)	66.2 (53.2, 77.8)	55.9 (43.6, 67.5)
Shizuoka	48.1	52.1	44.3	54.4	56.8	51.8	58.1 (47.0, 68.5)	59.8 (49.6, 68.9)	56.1 (42.2, 68.7)
Aichi	40.9	46.7	35.2	48.5	52.3	44.9	54.7 (43.1, 65.8)	56.7 (48.6, 64.5)	53.1 (38.8, 66.7)
Mie	40.4	46.6	34.5	48.7	52.4	45.1	55.6 (41.8, 69.3)	57.2 (46.7, 67.1)	53.8 (34.8, 72.0)
Shiga	39.6	46.9	33.1	47.6	52.7	43.6	55.0 (37.8, 72.0)	58.1 (38.6, 75.7)	52.8 (35.6, 69.7)
Kyoto	37.8	43.5	32.8	42.0	45.5	38.6	46.9 (33.0, 61.8)	49.8 (28.7, 70.7)	43.6 (32.8, 54.6)
Osaka	32.3	37.4	27.9	42.2	46.6	38.4	51.2 (38.1, 64.6)	54.4 (42.4, 66.3)	48.7 (33.0, 65.5)
Hyogo	37.0	44.8	30.1	44.2	48.0	40.7	50.7 (38.9, 62.2)	52.4 (35.6, 68.9)	49.3 (40.0, 58.6)
Nara	35.5	43.2	29.7	44.0	52.0	37.6	52.1 (35.3, 67.9)	58.9 (47.7, 69.1)	46.2 (27.9, 65.0)
Wakayama	40.0	44.7	35.9	46.5	48.9	44.6	50.7 (40.3, 61.0)	52.3 (44.1, 60.3)	49.9 (39.0, 61.1)
Tottori	48.7	51.4	45.8	56.3	58.5	55.4	62.0 (53.6, 70.1)	63.8 (47.7, 77.8)	61.0 (52.3, 69.0)
Shimane	47.8	51.1	43.8	55.8	56.6	53.8	62.6 (45.0, 78.9)	62.5 (35.9, 83.0)	61.5 (47.4, 73.6)
Okayama	52.1	54.2	49.7	57.7	59.1	56.3	60.8 (54.0, 67.3)	62.3 (55.6, 68.8)	59.9 (53.1, 66.6)
Hiroshima	41.3	46.3	36.9	47.7	54.3	41.5	52.3 (43.7, 60.6)	59.0 (48.6, 68.5)	45.7 (36.9, 55.3)
Yamaguchi	40.9	47.1	35.2	45.5	51.6	39.0	48.8 (41.8, 56.3)	54.4 (46.4, 62.2)	42.3 (34.9, 49.8)
Tokushima	39.5	43.6	36.1	46.4	50.7	43.3	52.3 (40.8, 63.8)	56.4 (41.2, 70.8)	49.4 (39.7, 59.3)
Kagawa	46.3	51.1	42.6	54.0	56.9	50.5	60.3 (35.9, 81.3)	61.2 (31.2, 85.1)	57.7 (32.6, 79.6)
Ehime	43.4	46.9	40.1	48.4	53.1	43.7	52.6 (45.0, 60.1)	58.7 (42.8, 73.3)	47.1 (36.5, 58.0)
Kochi	46.5	47.9	44.6	56.5	58.7	56.1	63.7 (50.0, 76.4)	65.6 (54.6, 75.4)	63.9 (48.6, 77.6)
Fukuoka	36.2	41.6	31.5	44.4	48.8	40.4	51.6 (38.1, 65.3)	54.8 (42.4, 66.6)	48.4 (33.6, 63.4)
Saga	45.3	49.7	42.2	54.4	58.4	50.6	59.8 (52.8, 66.2)	62.1 (45.5, 77.1)	56.3 (48.7, 63.9)
Nagasaki	37.3	43.0	32.3	44.9	47.4	42.6	50.5 (43.9, 57.1)	50.0 (43.7, 56.5)	51.2 (39.3, 62.6)
Kumamoto	47.1	49.6	44.9	52.8	54.7	51.1	57.5 (14.9, 90.6)	59.3 (7.1, 96.5)	55.7 (28.9, 79.7)
Oita	41.8	47.2	37.8	49.8	53.4	47.8	57.1 (33.7, 78.2)	59.3 (35.9, 79.9)	56.3 (32.9, 77.1)
Miyazaki	42.1	46.1	38.5	50.4	54.5	48.0	56.7 (43.7, 69.2)	60.5 (50.9, 69.6)	55.0 (40.5, 68.5)
Kagoshima	46.4	49.2	43.1	51.4	54.4	48.0	56.4 (31.5, 78.9)	58.6 (34.9, 78.7)	53.7 (22.5, 82.3)
Okinawa	40.8	42.9	38.9	44.5	44.9	44.3	47.2 (39.0, 55.6)	47.1 (36.8, 57.5)	47.7 (40.5, 54.8)

Data from the Ministry of Health, Labour and Welfare Comprehensive Survey of Living Conditions via e-Stat Japan. Coverage rates for adults aged 40–69 years (2013–2022). Pre-2013 survey waves were excluded due to a structural break in screening trends following the 2012 Cancer Control Plan revision. Projections derived from Bayesian logit regression models with 95% credible intervals (CrI). M–F diff = male–female difference in screening coverage (percentage points).

**Table 2:** Projected trends and target achievement for lung cancer screening by prefecture and sex.

	Total			Men			Women		
Prefecture	APC ^1^	P(60%) ^2^	Target Year ^3^	APC ^1^	P(60%) ^2^	Target Year ^3^	APC ^1^	P(60%) ^2^	Target Year ^3^
Nationwide	0.83 (−0.05, 1.67)	0.113 (0.063, 0.163)	2040	0.64 (−0.35, 1.55)	0.254 (0.204, 0.304)	2040	1.01 (0.11, 1.93)	0.070 (0.020, 0.120)	2039
Hokkaido	0.55 (−0.42, 1.46)	0.016	2080	0.52 (−0.65, 1.63)	0.050	2075	0.56 (−0.39, 1.48)	0.012	2086
Aomori	1.23 (−0.42, 2.76)	0.802	2028 †	0.96 (−0.96, 2.84)	0.790	2029 †	1.34 (0.17, 2.36)	0.794	2029 †
Iwate	0.71 (−0.22, 1.60)	0.877	2026 †	0.64 (−1.34, 2.69)	0.782	2032	0.78 (0.01, 1.57)	0.840	2028 †
Miyagi	0.59 (−0.81, 2.01)	0.846	2024 †	0.32 (−0.91, 1.60)	0.888	2015 †	0.70 (−1.13, 2.45)	0.707	2036
Akita	0.57 (−0.75, 1.89)	0.521	2042	0.55 (−0.87, 1.93)	0.744	2033	0.75 (−0.64, 2.15)	0.455	2039
Yamagata	0.99 (0.09, 1.88)	0.991	2014 †	0.82 (−0.37, 2.08)	0.981	2013 †	1.18 (−0.05, 2.47)	0.981	2016 †
Fukushima	0.64 (−0.38, 1.66)	0.564	2034	0.45 (−0.19, 1.07)	0.843	2027 †	0.76 (−0.79, 2.22)	0.346	2044
Ibaraki	0.64 (−1.24, 2.53)	0.267	2091	0.48 (−1.54, 2.50)	0.365	—	0.77 (−1.20, 2.54)	0.197	2063
Tochigi	0.55 (−0.86, 1.98)	0.273	2064	0.37 (−0.96, 1.68)	0.346	—	0.66 (−0.74, 1.98)	0.178	2053
Gunma	0.84 (−0.92, 2.54)	0.694	2033	0.70 (−1.12, 2.60)	0.728	2037	0.95 (−0.93, 2.63)	0.595	2035
Saitama	0.68 (−0.66, 1.95)	0.071	2060	0.33 (−1.00, 1.55)	0.069	—	1.02 (−0.23, 2.13)	0.071	2044
Chiba	0.75 (−0.24, 1.76)	0.214	2039	0.58 (−0.26, 1.39)	0.292	2038	0.92 (−0.11, 1.95)	0.170	2038
Tokyo	1.16 (−0.02, 2.28)	0.377	2034	0.78 (−0.49, 1.93)	0.381	2037	1.48 (0.21, 2.56)	0.407	2032
Kanagawa	0.87 (0.14, 1.54)	0.072	2037	0.56 (−0.29, 1.33)	0.235	2039	1.16 (0.49, 1.79)	0.039	2036
Niigata	0.62 (−0.86, 2.12)	0.850	2024 †	0.43 (−1.20, 2.20)	0.852	2016 †	0.77 (−0.44, 1.92)	0.810	2028 †
Toyama	0.80 (0.12, 1.49)	0.903	2026 †	0.51 (−0.32, 1.38)	0.880	2025 †	1.03 (0.22, 1.82)	0.879	2027 †
Ishikawa	0.81 (−0.64, 2.18)	0.597	2035	0.71 (−1.41, 2.81)	0.737	2038	0.88 (−0.22, 1.87)	0.240	2037
Fukui	0.43 (−0.24, 1.08)	0.041	2055	0.24 (−0.48, 0.96)	0.075	—	0.73 (0.07, 1.43)	0.051	2041
Yamanashi	0.94 (0.20, 1.70)	0.980	2019 †	0.67 (−0.34, 1.76)	0.959	2017 †	1.15 (0.27, 2.06)	0.970	2022 †
Nagano	0.72 (−0.08, 1.50)	0.737	2030 †	0.66 (−0.38, 1.73)	0.885	2024 †	0.82 (−0.06, 1.66)	0.345	2034
Gifu	1.24 (0.07, 2.30)	0.593	2031	1.33 (0.07, 2.56)	0.916	2025 †	1.22 (0.25, 2.14)	0.143	2035
Shizuoka	0.61 (−0.37, 1.57)	0.272	2040	0.48 (−0.40, 1.42)	0.502	2038	0.71 (−0.46, 1.85)	0.168	2045
Aichi	0.84 (−0.18, 1.78)	0.094	2042	0.63 (−0.11, 1.37)	0.110	2041	1.04 (−0.23, 2.16)	0.090	2042
Mie	0.91 (−0.35, 2.10)	0.167	2041	0.67 (−0.33, 1.61)	0.195	2041	1.08 (−0.49, 2.43)	0.150	2042
Shiga	0.87 (−0.72, 2.32)	0.184	2048	0.64 (−1.13, 2.33)	0.377	2058	1.12 (−0.29, 2.33)	0.111	2042
Kyoto	0.49 (−0.80, 1.72)	0.032	—	0.33 (−1.60, 2.17)	0.095	—	0.60 (−0.37, 1.42)	0.011	2073
Osaka	1.10 (−0.01, 2.10)	0.057	2042	1.03 (−0.01, 1.96)	0.091	2039	1.12 (−0.19, 2.13)	0.045	2044
Hyogo	0.82 (−0.24, 1.90)	0.049	2050	0.42 (−1.25, 1.97)	0.104	—	1.11 (0.41, 1.80)	0.021	2040
Nara	1.00 (−0.42, 2.22)	0.090	2045	1.06 (0.12, 2.00)	0.367	2033	0.90 (−0.82, 2.25)	0.054	2058
Wakayama	0.64 (−0.28, 1.46)	0.028	2055	0.46 (−0.22, 1.10)	0.022	2062	0.84 (−0.04, 1.63)	0.024	2048
Tottori	0.86 (0.07, 1.70)	0.799	2029 †	0.76 (−0.83, 2.30)	0.806	2028 †	0.99 (0.18, 1.78)	0.688	2030 †
Shimane	0.91 (−0.69, 2.50)	0.719	2032	0.68 (−1.69, 2.97)	0.688	2062	1.09 (−0.26, 2.32)	0.678	2031
Okayama	0.63 (0.00, 1.26)	0.696	2030 †	0.59 (−0.02, 1.28)	0.874	2027 †	0.70 (0.04, 1.34)	0.481	2032
Hiroshima	0.75 (0.03, 1.42)	0.027	2045	0.91 (−0.02, 1.84)	0.368	2034	0.54 (−0.22, 1.23)	0.009	2071
Yamaguchi	0.48 (−0.13, 1.12)	0.011	2068	0.44 (−0.33, 1.18)	0.048	2058	0.41 (−0.27, 1.06)	0.005	—
Tokushima	0.82 (−0.13, 1.66)	0.043	2045	0.89 (−0.56, 2.23)	0.219	2043	0.83 (0.02, 1.57)	0.020	2047
Kagawa	0.76 (−1.66, 3.04)	0.543	2064	0.56 (−1.76, 3.09)	0.610	—	0.83 (−1.45, 2.91)	0.393	2058
Ehime	0.58 (−0.14, 1.28)	0.028	2051	0.71 (−0.70, 2.04)	0.383	2042	0.46 (−0.53, 1.38)	0.018	2089
Kochi	1.09 (−0.13, 2.24)	0.841	2028 †	1.13 (−0.01, 2.21)	0.916	2025 †	1.19 (−0.28, 2.57)	0.811	2028 †
Fukuoka	0.89 (−0.32, 1.96)	0.060	2047	0.79 (−0.37, 1.85)	0.115	2044	0.95 (−0.24, 1.98)	0.040	2049
Saga	1.01 (0.40, 1.62)	0.490	2031	0.96 (−0.45, 2.43)	0.709	2031	0.88 (0.18, 1.57)	0.087	2036
Nagasaki	0.83 (0.29, 1.36)	0.013	2044	0.48 (−0.09, 1.04)	0.011	2062	1.10 (0.16, 1.92)	0.042	2041
Kumamoto	0.67 (−2.67, 3.69)	0.386	—	0.62 (−2.97, 3.95)	0.529	—	0.70 (−1.57, 2.63)	0.169	—
Oita	0.85 (−1.21, 2.74)	0.343	2054	0.67 (−1.41, 2.71)	0.483	2069	1.04 (−0.88, 2.79)	0.301	2044
Miyazaki	0.88 (−0.28, 1.99)	0.203	2039	0.91 (−0.01, 1.75)	0.595	2031	0.97 (−0.19, 2.06)	0.132	2040
Kagoshima	0.50 (−1.67, 2.69)	0.319	—	0.49 (−1.53, 2.54)	0.436	—	0.47 (−2.29, 3.08)	0.265	—
Okinawa	0.36 (−0.41, 1.10)	0.011	—	0.21 (−0.70, 1.07)	0.014	—	0.54 (−0.10, 1.18)	0.010	2064

^1^ APC = annual percentage change in coverage (percentage points per year) with 95% credible interval. ^2^ P(60%) = probability of achieving ≥60% coverage by 2028, based on Bayesian posterior distribution. The 60% benchmark is the *Health Japan 21* target. ^3^ Target year = first calendar year in which the posterior probability of reaching 60% coverage exceeds 0.80. Years beyond 2028 represent linear extrapolations of the fitted post-2013 trend and should be interpreted as the year at which the *current trajectory* would cross the 60% threshold, not as point forecasts. — = posterior probability does not exceed 0.80 by 2100. † Indicates that the target year is at or before 2030, i.e., the prefecture–sex unit is on track to meet the 60% target around the 2028 horizon. Bayesian logit regression models using prefecture-level surveillance data (2013–2022).

## Data Availability

The original data presented in the study are openly available from the Portal Site of the Official Statistics of Japan website (eStat): https://www.e-stat.go.jp/.

## Supplementary Materials

The following supporting information can be downloaded at https://www.mdpi.com/article/10.3390/cancers18101498/s1: Table S1: Out-of-sample model validation: training 2013–2019, validation 2022; Figure S1: Observed versus predicted lung cancer screening uptake in 2022 from out-of-sample validation models trained on 2013 to 2019 data; Figure S2: Prefecture-level out-of-sample validation performance of lung cancer screening uptake models trained on 2013 to 2019 data and evaluated against observed 2022 values, shown separately for women and men.
