# Supplementary material for: Progress and Disparities in Lung Cancer Screening in Japan: A Bayesian Analysis Toward Achieving Health Japan 21 Targets
Source: Cancers (Basel). 2026 May 7;18(10):1498. doi: 10.3390/cancers18101498 (PMC13204297; doi:10.3390/cancers18101498)
Supplement: Supplementary file 1 [file cancers-18-01498-s001.zip › Table_S1.pdf]

Supplementary Table S1. Out-of-sample model validation: training 2013–2019, validation 2022

Group	n[1]	MAE[2]	ME[3]	RMSE[4]	MAPE[5]	Coverage[6]
Men	46	3.50	2.96	3.50	6.5	100.0%
Total Population	46	3.35	3.22	3.35	6.7	100.0%
Women	46	3.55	3.27	3.55	7.6	100.0%
**Overall**	**138**	**3.47**	**3.15**	**3.47**	**6.9**	**100.0%**

[1]Number of prefecture-gender combinations with sufficient data for validation

[2]Mean Absolute Error: average absolute difference between predicted and observed values (percentage points)

[3]Mean Error: average bias between predicted and observed values (percentage points); positive values indicate overestimation.

[4]Root Mean Square Error: square root of average squared differences (percentage points).

[5]Mean Absolute Percentage Error: average absolute percentage difference relative to observed values.

[6]Proportion of observed values falling within 95% credible intervals of predictions

Models trained on 2013–2019 data (3 post-structural-break survey waves), validated against observed 2022 values. Pre-2013 data excluded due to policy-driven structural break.
