# Supplementary figures and images for: Progress and Disparities in Lung Cancer Screening in Japan: A Bayesian Analysis Toward Achieving Health Japan 21 Targets
Source: Cancers (Basel). 2026 May 7;18(10):1498. doi: 10.3390/cancers18101498 (PMC13204297; doi:10.3390/cancers18101498)

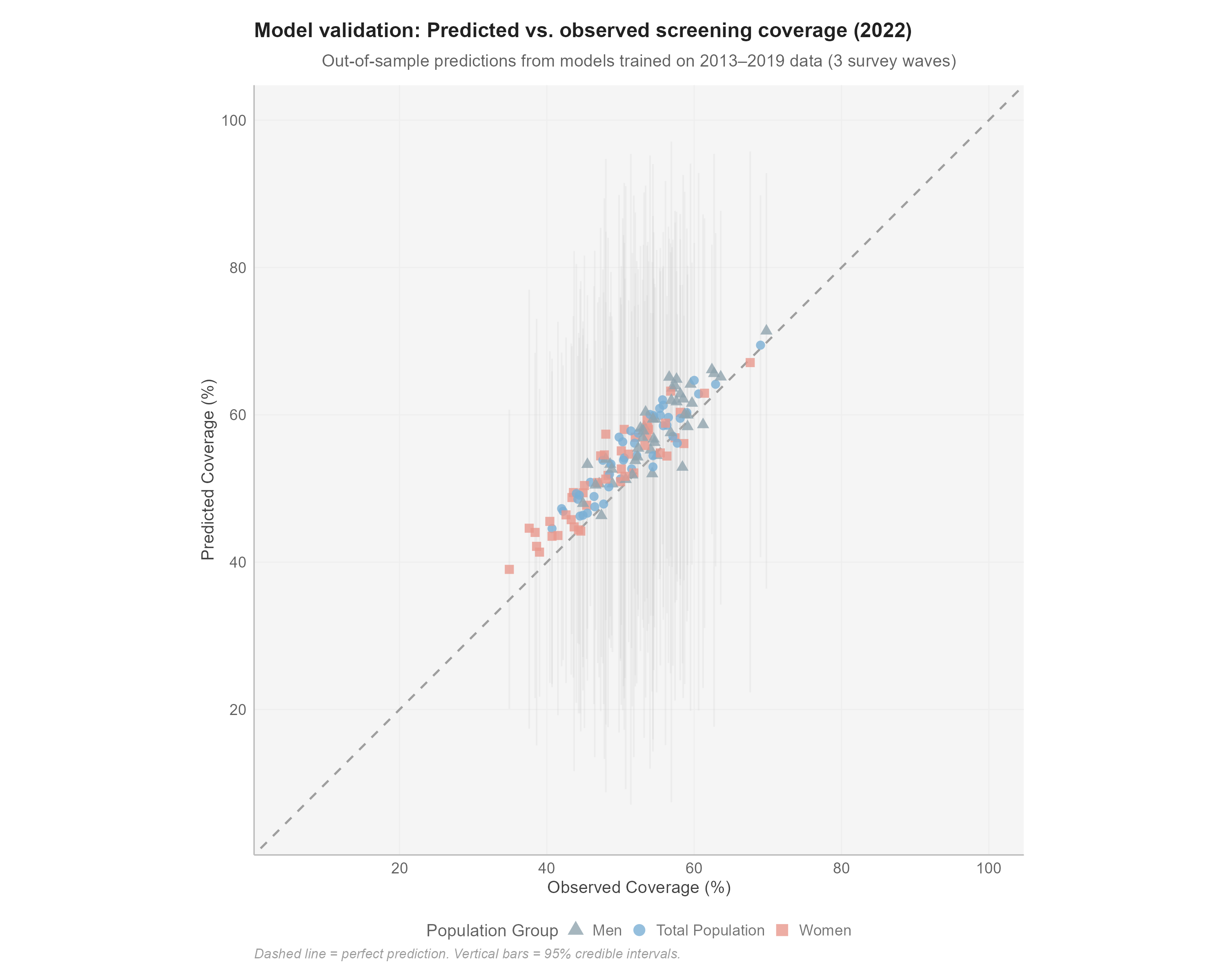

Supplement: Supplementary file 1 [file cancers-18-01498-s001.zip › Figure_S1.tiff]

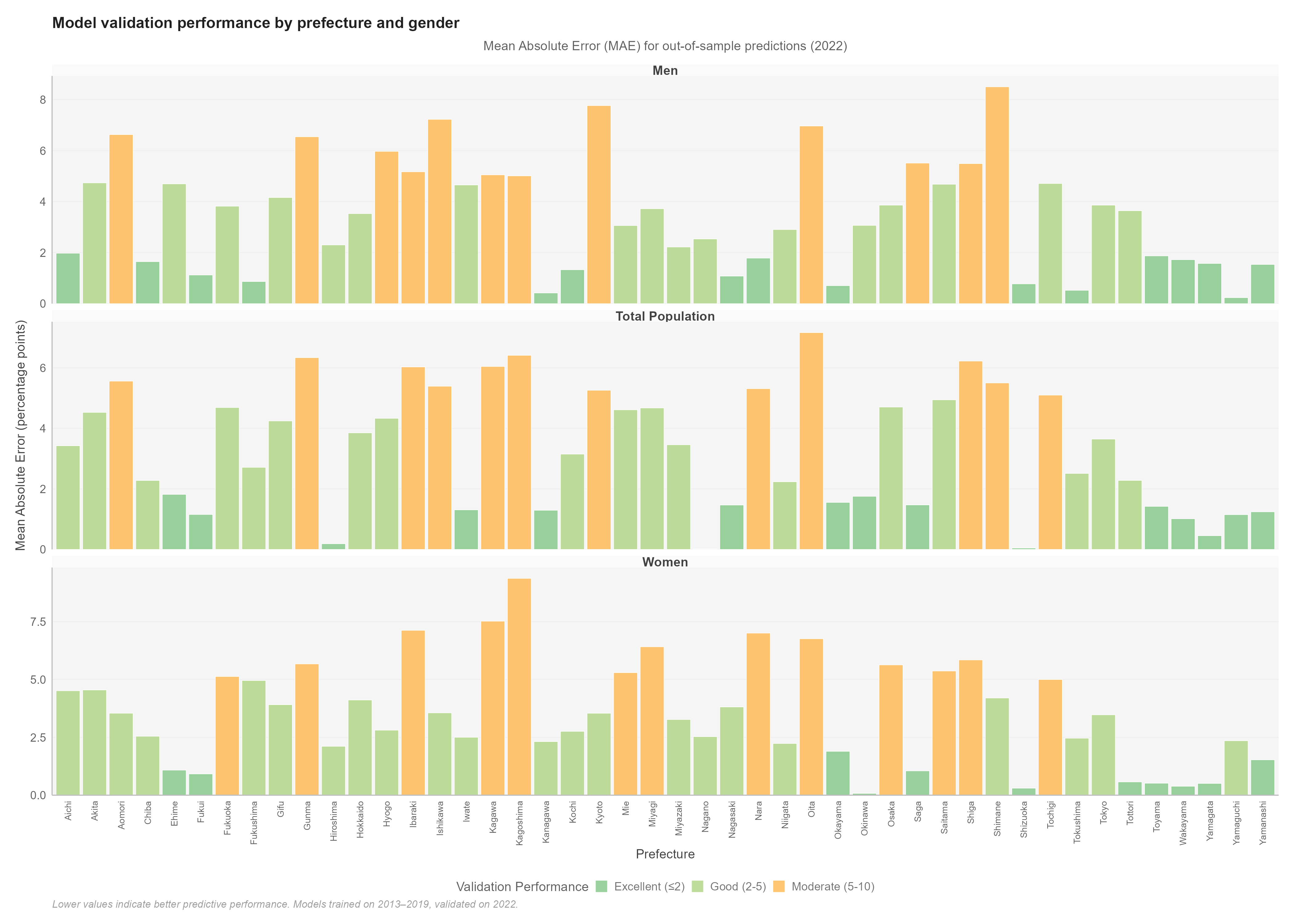

Supplement: Supplementary file 1 [file cancers-18-01498-s001.zip › Figure_S2.tiff]
